# Supplementary material for: Antigen-Specific IFN-γ/IL-17-Co-Producing CD4+ T-Cells are the Determinants for Protective Efficacy of Tuberculosis Subunit Vaccine
Source: Vaccines (Basel). 2020 Jun 11;8(2):300. doi: 10.3390/vaccines8020300 (PMC7350228; doi:10.3390/vaccines8020300)
Supplement: Supplementary file 1 [file vaccines-08-00300-s001.pdf]

Antigen-specific IFN-γ/IL-17-co-producing $CD4^+$ T-cells are the determinants for protective efficacy of tuberculosis subunit vaccine

Han-Gyu Choi[1,3], Kee Woong Kwon[2,3], Seunga Choi[1], Yong Woo Back[1], Hye-Soo Park[1], Soon Myung Kang[2], Eunsol Choi[2], Sung Jae Shin[2*] & Hwa-Jung Kim[1*]

[1]Department of Microbiology, and Medical Science, College of Medicine, Chungnam National University, Daejeon, Republic of Korea

[2]Department of Microbiology, Institute for Immunology and Immunological Diseases, Brain Korea 21 PLUS Project for Medical Science, Yonsei University College of Medicine, Seoul, Republic of Korea

[3]These authors contributed equally.

[*]Corresponding authors

Hwa-Jung Kim, email: hjukim@cnu.ac.kr,

Sung Jae Shin, email: sjshin@yuhs.ac

Supplementary Information

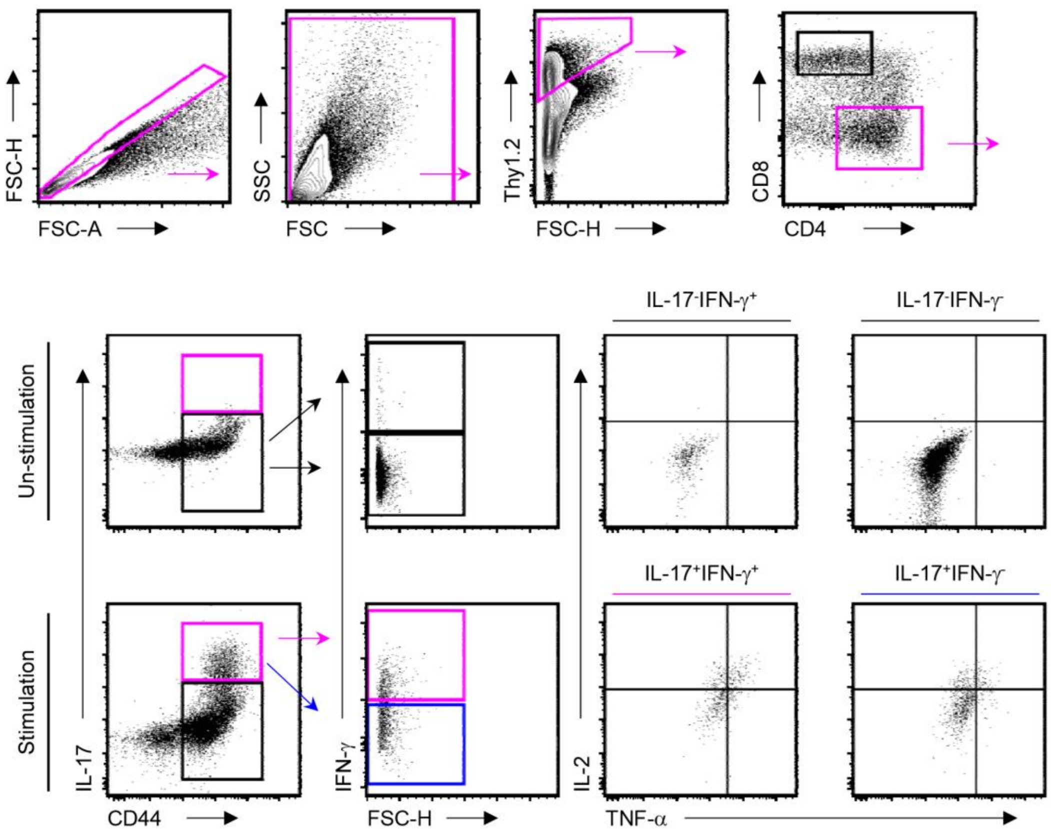

Supplementary Fig. 1. Gating strategy for the assessment of intracellular cytokines. All samples stained for surface and intracellular cytokines were gated based on forward scatter (FSC) and side scatter (SSC). T-cells were gated from lymphocytes by FSC vs. SSC on the basis of Thy1.2/CD4 expression for $CD4^{+}$ T-cells. To distinguish multifunctional T-cell subsets, gates indicating positive staining for each cytokine (IFN-γ, IL-2, IL-17A, and TNF-α) were delineated using the unstimulated control to determine background staining.~~Using a $Thy1.2^{+}CD4^{+}$ T-cell gate, specific staining for IFN-γ, IL-2, IL-17A, and TNF-α are shown for isotype-control and stimulated spleen and lung cells.~~

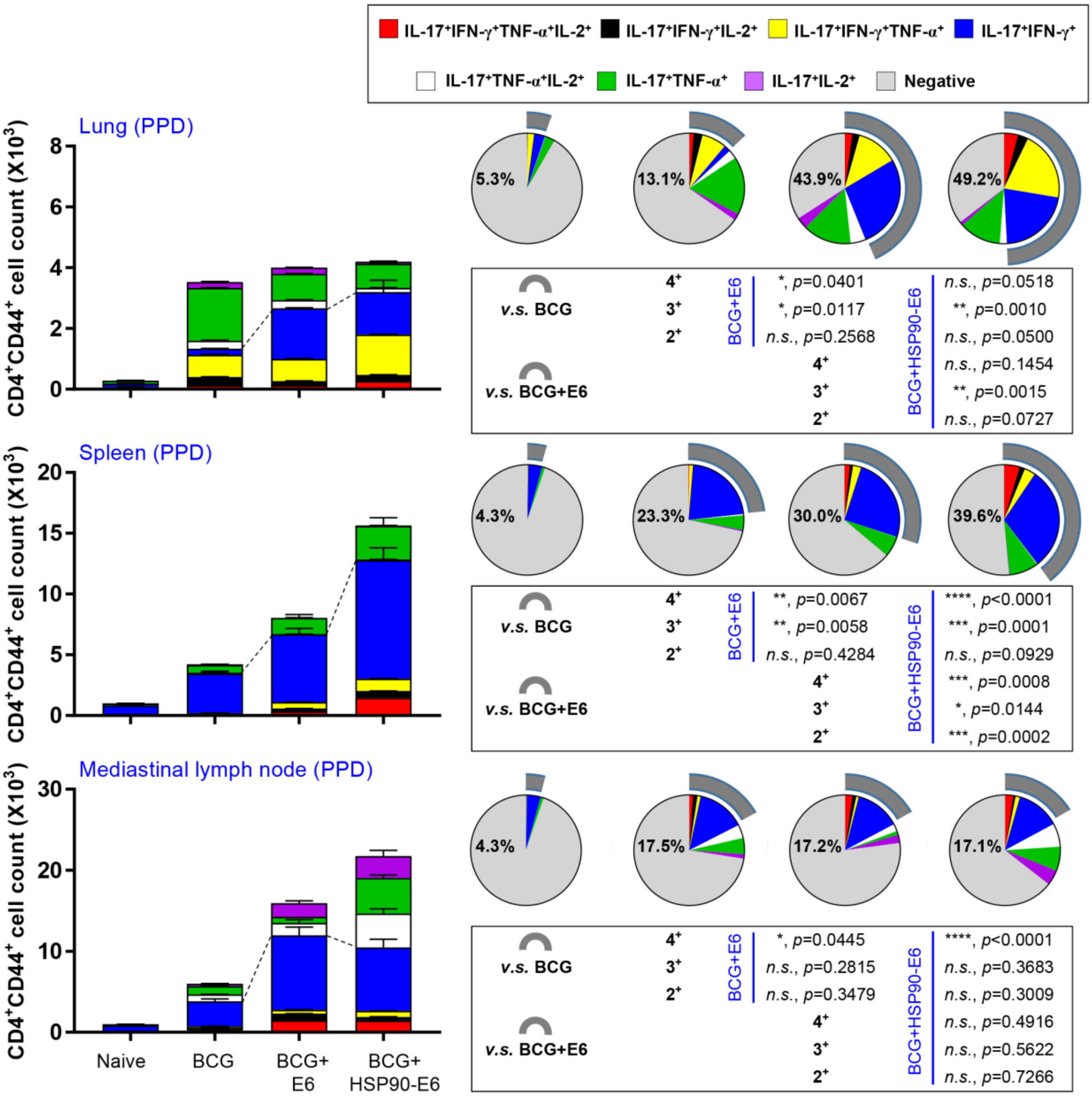

Supplementary Fig. 2. Ag-specific multifunctional T-cells are induced in the lungs, spleen, and lymph nodes in BCG+HSP90-E6-immunised mice. Mice were immunised and euthanised as described in the Methods section. Four weeks after the last immunisation, the mice were sacrificed and lung, spleen, and lymph-node cells collected from the mice were treated with PPD (2 μg/ml) at 37°C for 12 h in the presence of GolgiStop. Upon stimulation with PPD, cell counts of Ag-specific, multifunctional $CD4^{+}CD44^{+}$ T-cells producing IFN-γ, IL-17, and/or TNF-α, IL-2 in lung, spleen, and lymph-node cells from each immunised group were determined by flow cytometry. Gray arc denotes the percentage of cytokine-positive T-cells

(IL-17$^+$IFN-γ^+TNF-α^+IL-2$^+$-, IL-17$^+$IFN-γ^+IL-2$^+$-, IL-17$^+$IFN-γ^+TNF-α^+-, and IL-17$^+$IFN-γ^+-CD4$^+$CD44$^+$ T-cells). 2$^+$ stands for sum percentages of double-cytokine positive T-cells (IL-17$^+$IFN-γ^+, IL-17$^+$TNF-α^+, and IL-17$^+$IL-2$^+$), 3$^+$ stands for triple-cytokine positive T-cells (IL-17$^+$IFN-γ^+IL-2$^+$, IL-17$^+$IFN-γ^+TNF-α^+ and IL-17$^+$TNF-α^+IL-2$^+$), and 4$^+$ stands for quadruple-cytokine positive T-cells (IL-17$^+$IFN-γ^+TNF-α^+IL-2$^+$). Data are the mean ± SD for 5 mice from each group. *n.s.*: not significant, *$p < 0.05$, **$p < 0.01$, ***$p < 0.001$ and ****$p < 0.0001$ compared to BCG-immunised mice. *n.s.*: not significant, **$p < 0.01$ and ***$p < 0.001$ between BCG+ESAT-6- and BCG+HSP90-E6-immunised mice.

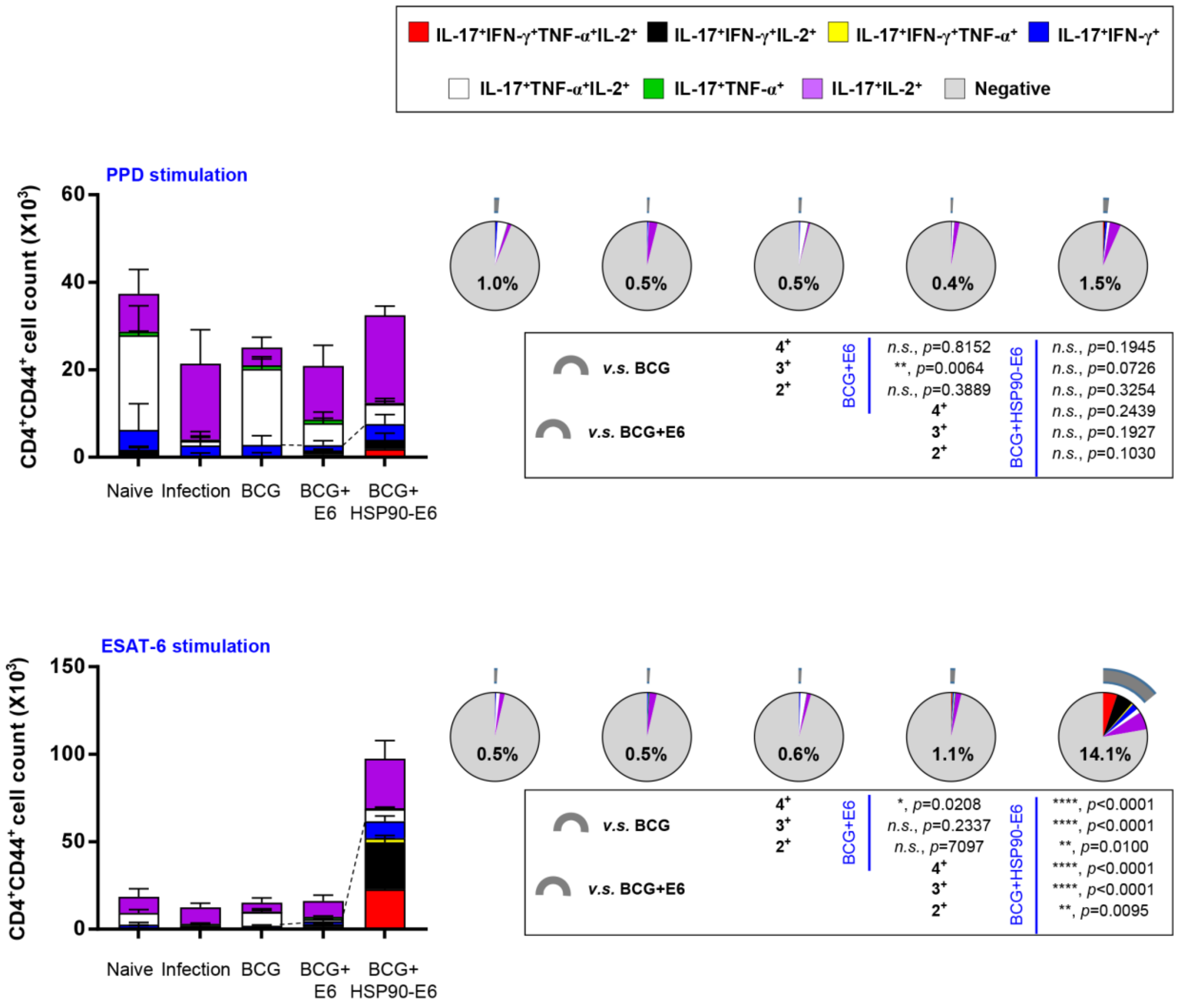

Supplementary Fig. 3. Induction of Ag-specific multifunctional T-cells is accompanied with the production of Th1/Th17-related cytokines after challenge with Mtb HN878. Mice in each group were sacrificed 10 weeks post-infection, and spleen cells were treated ESAT-6 (2 μg/ml) at 37°C for 12 h in the presence of GolgiStop. Upon stimulation with ESAT-6, cell counts of Ag-specific, multifunctional CD4$^+$CD44$^+$ T-cells producing IFN-γ, IL-17 and/or TNF-α and IL-2 in spleen cells from each immunised group were determined by flow cytometry. Gray arc denotes the percentage of cytokine-positive T-cells (IL-17$^+$IFN-γ^+TNF-α^+IL-2$^+$-, IL-17$^+$IFN-γ^+IL-2$^+$-, IL-17$^+$IFN-γ^+TNF-α^+-, and IL-17$^+$IFN-γ^+-CD4$^+$CD44$^+$ T-cells). 2$^+$ stands for sum percentages of double-cytokine positive T-cells (IL-17$^+$IFN-γ^+, IL-17$^+$TNF-α^+, and IL-17$^+$IL-2$^+$), 3$^+$ stands for triple-cytokine positive T-cells (IL-17$^+$IFN-γ^+IL-2$^+$, IL-17$^+$IFN-γ^+TNF-α^+

and IL-17$^+$TNF-α$^+$IL-2$^+$), and 4$^+$ stands for quadruple-cytokine positive T-cells (IL-17$^+$IFN-γ$^+$TNF-α$^+$IL-2$^+$). Data the mean ± SD for 7 mice from each group. *n.s.*: not significant, *$p < 0.05$, **$p < 0.01$ and ****$p < 0.0001$ compared to BCG-immunised mice. *n.s.*: not significant, **$p < 0.01$ and ****$p < 0.0001$ between BCG+ESAT-6- and BCG+HSP90-E6-immunised mice.

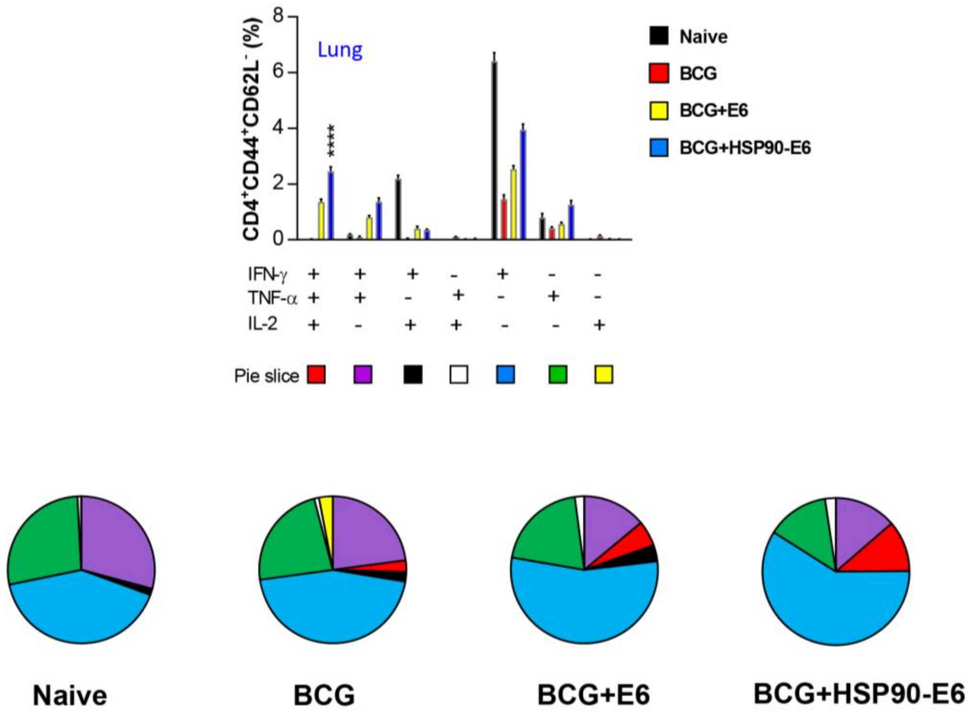

Supplementary Fig. 4. Ag-specific multifunctional T-cells are induced in the lungs in BCG+HSP90-E6-immunised mice. Mice were immunised and euthanised as described in the Methods section. Four weeks after the last immunisation, mice were sacrificed, and lungs cells were treated with ESAT-6 (2 μg/ml) at 37 °C for 12 h in the presence of GolgiStop. Upon stimulation with PPD, cell counts of Ag-specific, multifunctional $CD4^+CD44^+$ T-cells producing IFN-γ and/or TNF-α and IL-2 in the lung cells from each immunised group were determined by flow cytometry. Data are the mean ± SD for 5 mice from each group. ****$p <$ 0.0001 compared to BCG-immunised mice.

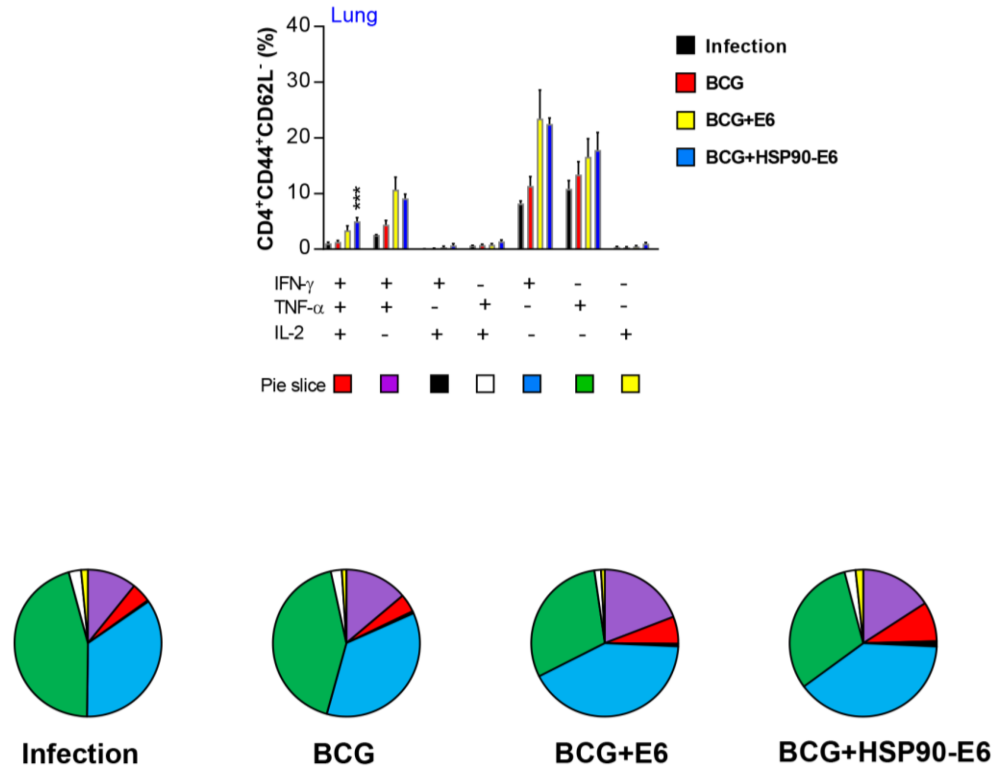

Supplementary Fig. 5. Induction of Ag-specific multifunctional T-cells accompanied with the production cytokines after challenge with Mtb HN878. Mice in each treatment group were sacrificed 10 weeks post-infection, and lung cells were treated with ESAT-6 (2 μg/ml) at 37°C for 12 h in the presence of GolgiStop. Upon stimulation with ESAT-6, cell counts of Ag-specific, multifunctional CD4$^+$CD44$^+$ T-cells producing IFN-γ and/or TNF-α and IL-2 in the lung cells from each immunised group were determined by flow cytometry. Data are the mean ± SD for 7 mice from each group. ***p < 0.001 compared to BCG-immunised mice.

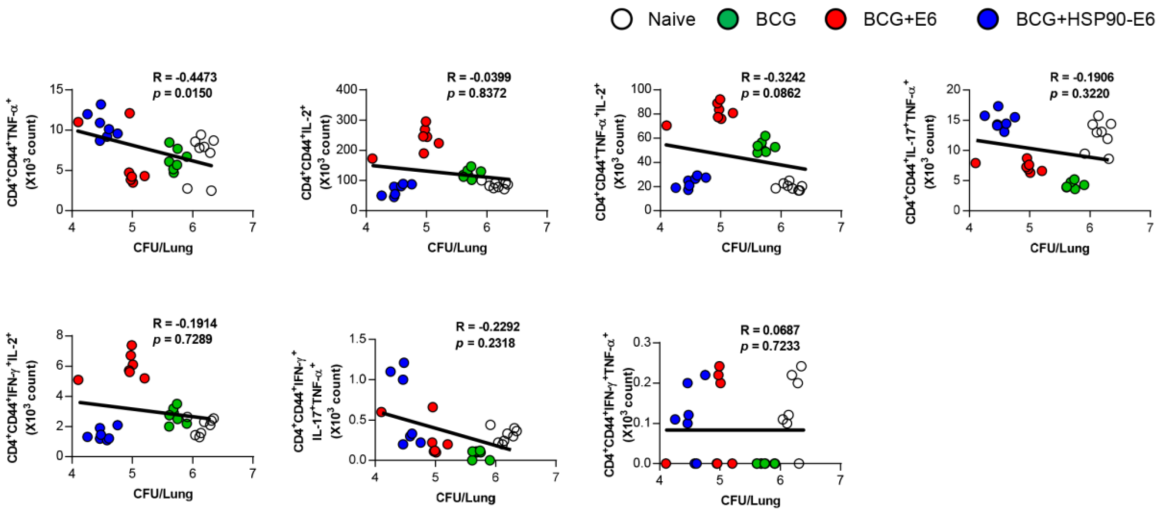

Supplementary Fig. 6. The protective correlation of protection with pre-infection driven immune response in the vaccinated and challenged mice. Relationship between protection (CFU) and ESAT-6 specific various cytokine combination in $CD4^{+}CD44^{+}$ T-cells is shown as a fitted regression line with the correlation coefficient. Spearman’s r and P values of the correlations are indicated. White circle: Naïve, green circle: BCG, red circle: BCG+E6, and blue circle: BCG+HSP90-E6.

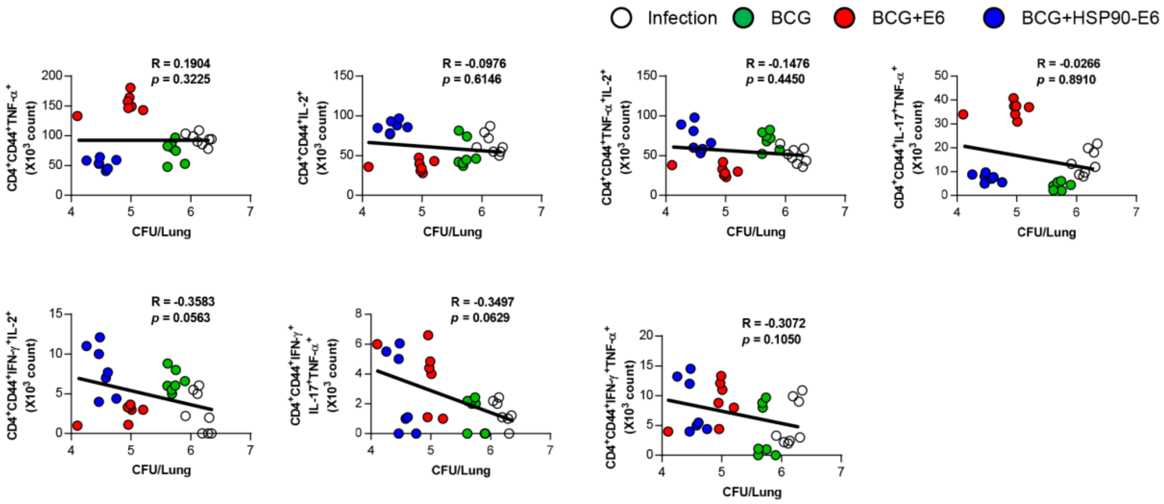

Supplementary Fig. 7. The protective correlation of protection with post-infection driven immune response in the vaccinated and challenged mice. Relationship between protection (CFU) and ESAT-6 specific various cytokine combination in $CD4^+CD44^+$ T-cells is shown as a fitted regression line with the correlation coefficient. Spearman's r and P values of the correlations are indicated. White circle: Infection, green circle: BCG, red circle: BCG+E6, and blue circle: BCG+HSP90-E6.

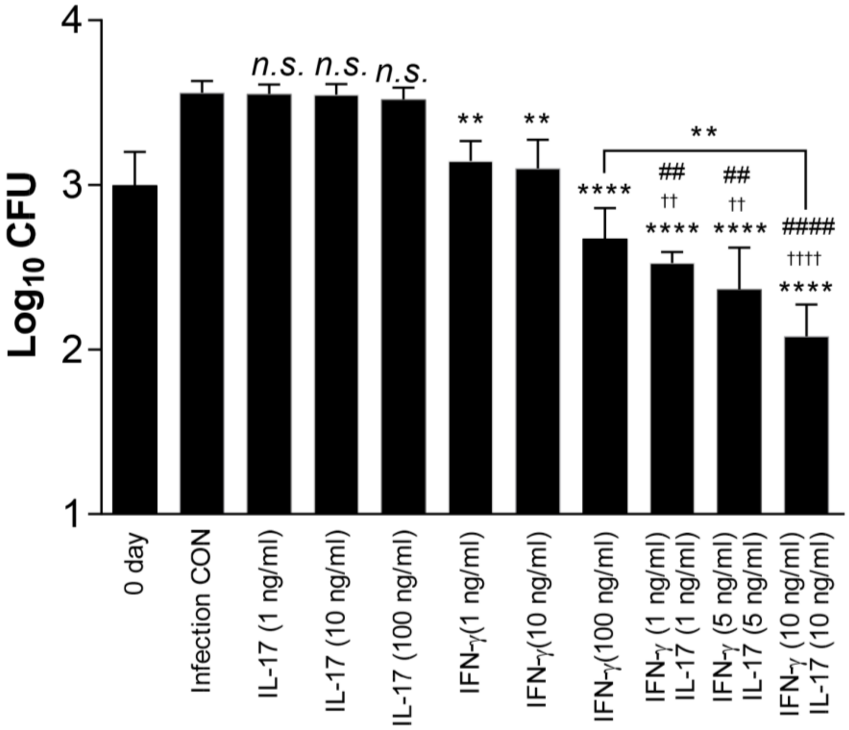

Supplementary Fig. 8. IFN-γ/IL-17 inhibits intracellular bacterial growth in Mtb-infected macrophages. Mtb-infected BMDMs were treated with IFN-γ (1 - 100 ng/ml), IL-17(1 - 100 ng/ml), or IFN-γ/IL-17 (1 - 10 ng/ml each) for 3 days. Intracellular Mtb growth in BMDMs was determined at time point 0 and 3 days after cytokine treatment. Data are the mean ± SD (*n* = 3); $^{**}p < 0.01$, or $^{****}p < 0.0001$ versus infection control, $^{\dagger\dagger}p < 0.01$ or $^{\dagger\dagger\dagger\dagger}p < 0.0001$ for co-treated vs. IFN-γ, $^{\#\#}p < 0.01$ or $^{\#\#\#\#\#}p < 0.0001$ co-treated vs. IFN-γ determined by one-way ANOVA. n.s.: no significant difference.

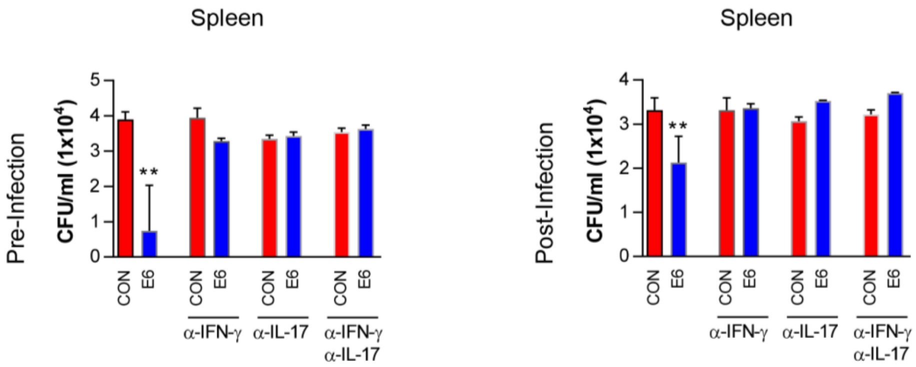

Supplementary Fig. 9. IFN-γ/IL-17 from supernatants of spleen cells from HSP90-E6-vaccinated mice inhibit intracellular Mtb growth. Mtb-infected BMDMs were treated with supernatants of ESAT-6-re-stimulated spleen cells from BCG+HSP90-E6-vaccinated mice in the presence of absence of anti-IFN-γ or anti-IL-17 for 3 days. Intracellular Mtb growth in BMDMs was determined on day 3. Data are the mean ± SD (n = 3); **p < 0.01, or ***p < 0.001.

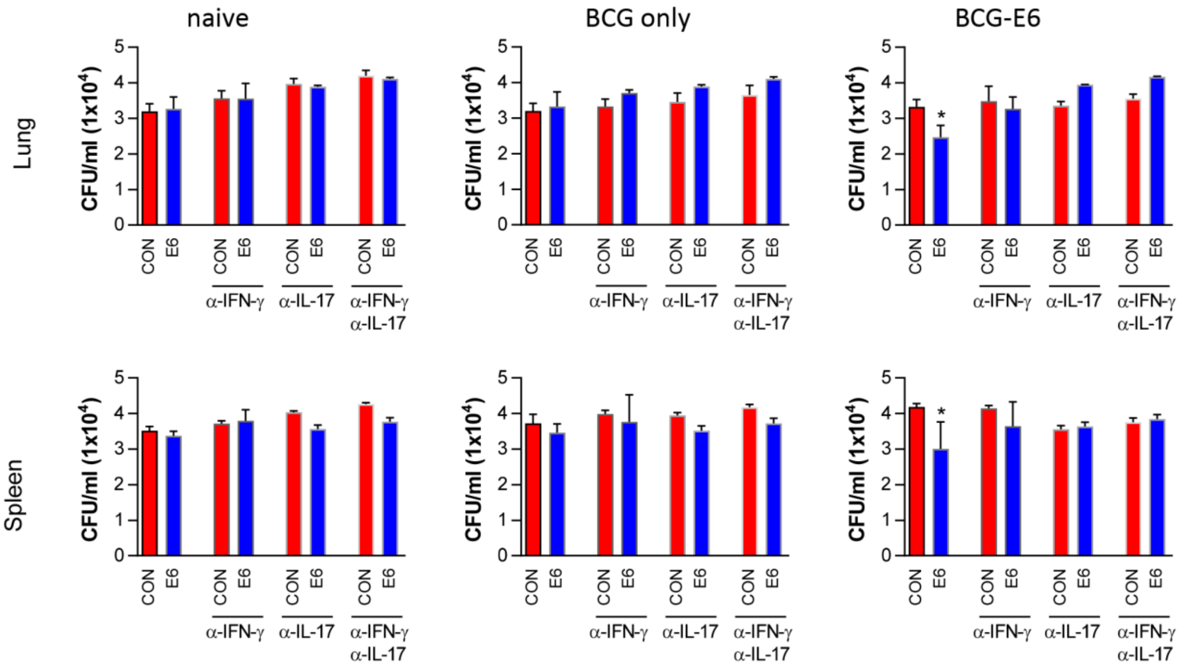

Supplementary Fig. 10. IFN-γ/IL-17 from supernatants of lung and spleen cells from ESAT-6-vaccinated mice inhibit intracellular Mtb growth. Mtb-infected BMDMs were treated with supernatants of ESAT-6-re-stimulated lung and spleen cells from BCG+E6-vaccinated mice in the presence of absence of anti-IFN-γ or anti-IL-17 for 3 days. Intracellular Mtb growth in BMDMs was determined on day 3. Data are the mean ± SD ($n = 3$); $*p < 0.05$. G1: naïve, G3: BCG, G4: BCG+E6.

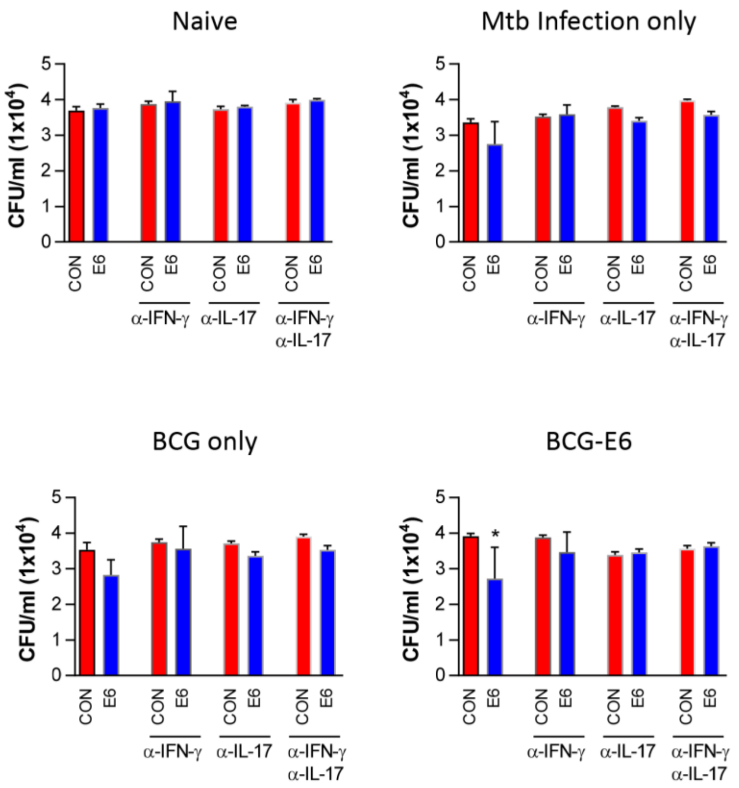

Supplementary Fig. 11. IFN-γ/IL-17 from supernatants of lung cells from infected ESAT-6-vaccinated mice inhibit intracellular Mtb growth. Mtb-infected BMDMs were treated with supernatants of ESAT-6-re-stimulated lung cells from BCG+E6-vaccinated mice in the presence of absence of anti-IFN-γ or anti-IL-17 for 3 days. Intracellular Mtb growth in BMDMs was determined on day 3. Data are the mean ± SD (n = 3); *p < 0.05. G1: naïve, G2: infection, G3: BCG, G4: BCG+E6.

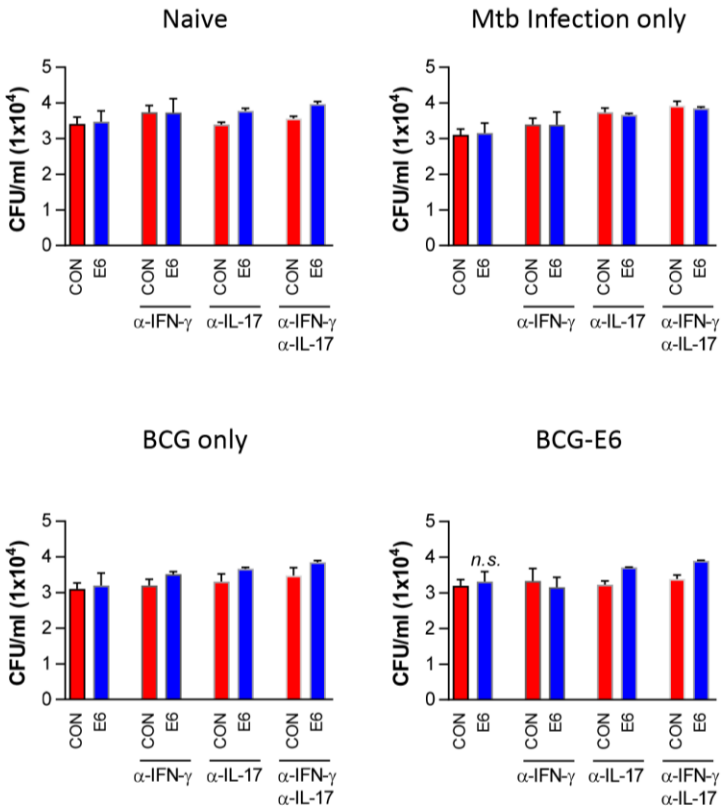

Supplementary Fig. 12. IFN-γ/IL-17 from supernatants of spleen cells from infected ESAT-6-vaccinated mice inhibit intracellular Mtb growth. Mtb-infected BMDMs were treated with supernatants of ESAT-6-re-stimulated spleen cells from BCG+E6-vaccinated mice in the presence of absence of anti-IFN-γ or anti-IL-17 for 3 days. Intracellular Mtb growth in BMDMs was determined on day 3. n.s.: no significant difference. G1: naïve, G2: infection, G3: BCG, G4: BCG+E6.

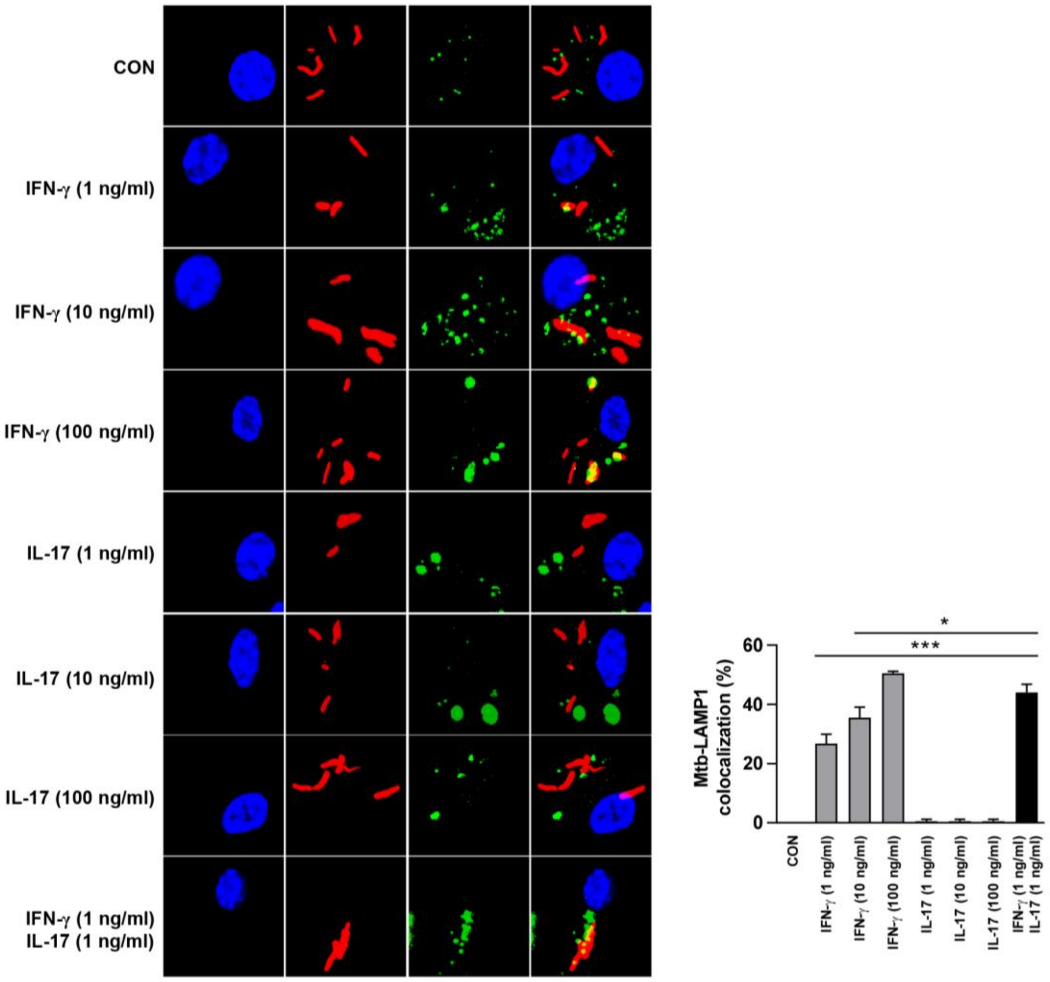

Supplementary Fig. 13. IFN-γ/IL-17 induces phagosome-lysosome fusion in Mtb-infected macrophages. BMDMs were infected with Mtb-RFP (MOI = 1) for 4 h, washed, incubated with/without IFN-γ (1 - 100 ng/ml), IL-17(1 - 100 ng/ml), or IFN-γ/IL-17 (1 ng/ml each) for 72 h, fixed with 4% paraformaldehyde, and immunolabeled with anti-LAMP1 antibody and Alexa 488-conjugated goat anti-rabbit or anti-rat IgG (green). Nuclei were counterstained with DAPI (blue). The cells were analysed by laser-scanning confocal microscopy. Scale bar, 10 μm. Quantification of Mtb–LAMP1 colocalisation is shown in the bar graph. Data are the mean ± SD of 50–100 cells per experiment (n = 3). $**p < 0.01$ and $***p < 0.001$ for treatment compared to infection-only controls (CON) or for difference between treatment data. *n.s.*, no significant difference.

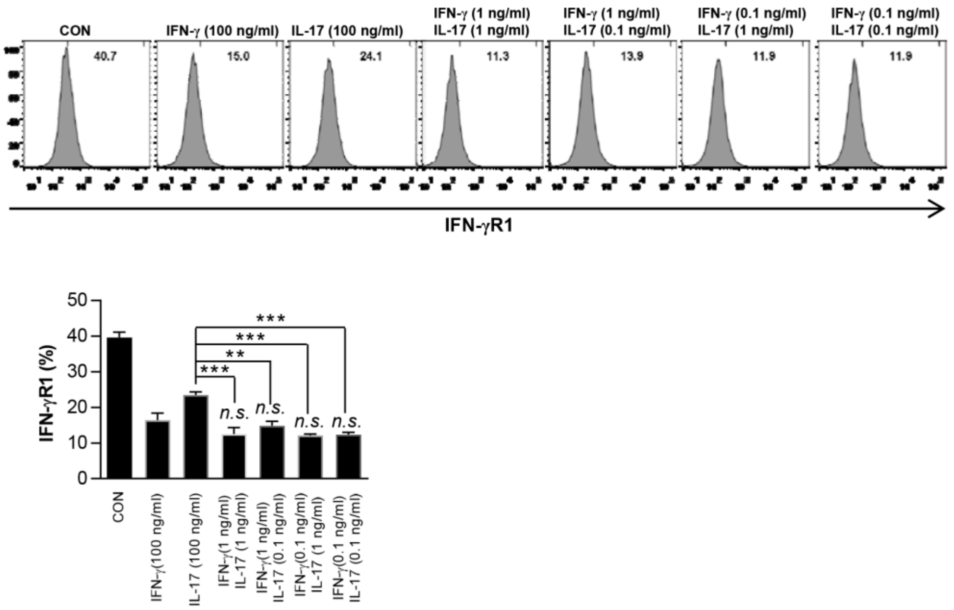

Supplementary Fig. 14. IFN-γ/IL-17 does not affect IFN-γR1 in Mtb-infected macrophages. BMDMs were infected with Mtb (MOI = 1) for 4 h, washed, incubated with/without IFN-γ (1 ng/ml), IL-17 (10 ng/ml), or IFN-γ/IL-17 (1 ng/ml each) for 72 h, immunolabeled with anti-IFN-γR1 antibody, and analysed by flow cytometry. Data are the mean ± SD (n = 5); *n.s.*: not significant, **$p < 0.01$ or ***$p < 0.0001$ versus infection control.

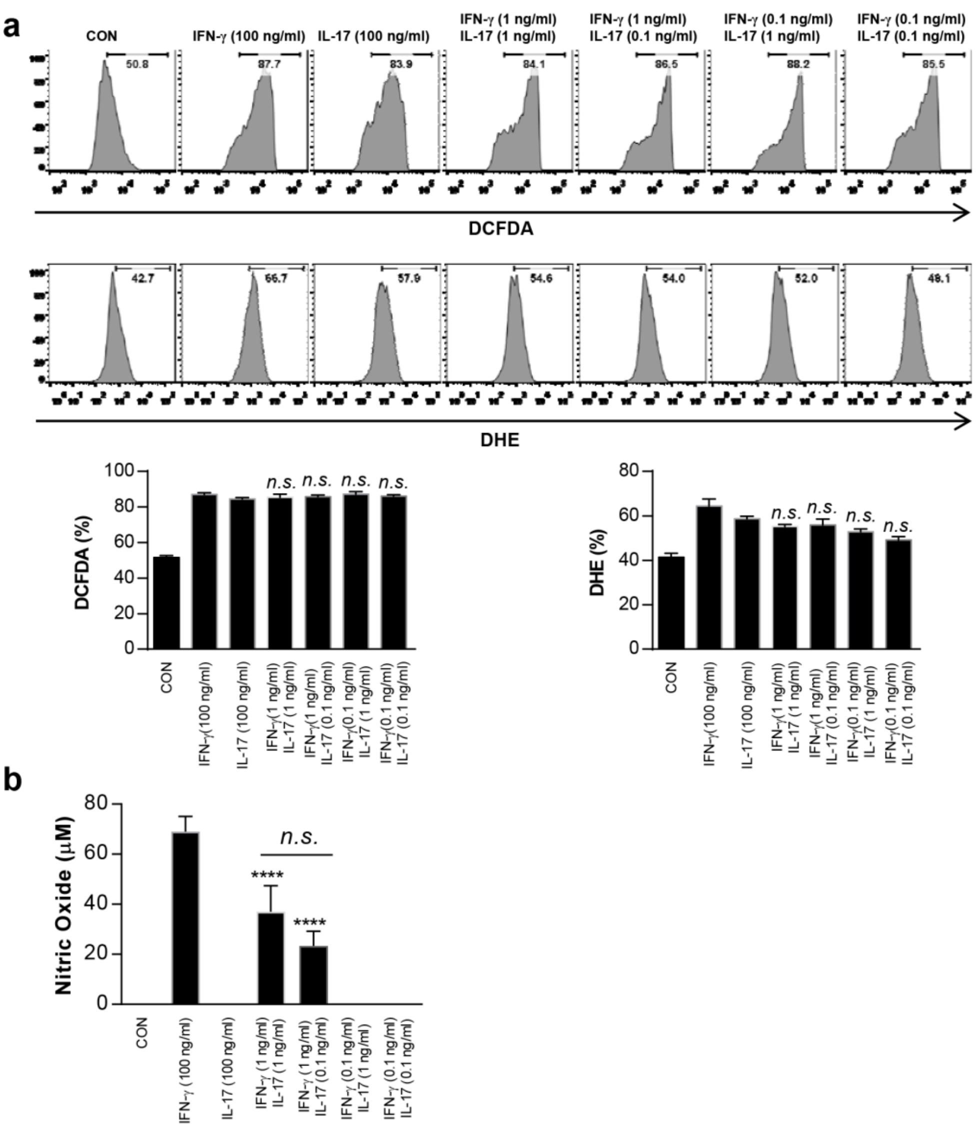

Supplementary Fig. 15. IFN-γ/IL-17 does not affect ROS or NO production in Mtb-infected macrophages. (**a**) BMDMs were infected with Mtb (MOI = 1) for 4 h, washed, incubated with/without IFN-γ (100 ng/ml), IL-17 (100 ng/ml), or IFN-γ/IL-17 (1 – 0.1 ng/ml each) for 72 h, and immunolabeled with anti- DCFDA or DHE antibodies, and analysed using flow cytometry. Data are the mean ± SD (*n* = 5); *n.s.*: not significant for IFN-γ/IL-17-treated vs. IFN-γ - or IL-17-treated cells, determined by one-way ANOVA. (**b**) NO production from culture supernatants were determined. Data are the mean ± SD (*n* = 5); *n.s.*: not significant,

****p < 0.0001 for IFN-γ/IL-17-treated vs. IFN-γ-treated cells, determined by one-way ANOVA.

	Variables of cytokine in T cells	Pre-infection		Variables of cytokine in T cells	Post-infection	
		Spearman r	P value		Spearman r	P value
Log_{10} CFU in Lung	IFN-γ^{+}TNF-α^{+}IL-2^{+}IL-17^{+}	-0.9082	*P* < 0.0001	IFN-γ^{+}IL-17^{+}	-0.7703	*P* < 0.0001
	IFN-γ^{+}IL-17^{+}	-0.8641	*P* < 0.0001	IFN-γ^{+}IL-2^{+}IL-17^{+}	-0.7133	*P* < 0.0001
	IL-17^{+}	-0.8183	*P* < 0.0001	IFN-γ^{+}TNF-α^{+}IL-2^{+}	-0.6845	*P* < 0.0001
	IFN-γ^{+}TNF-α^{+}IL-2^{+}	-0.6434	*P* = 0.0002	TNF-α^{+}IL-2^{+}IL-17^{+}	-0.5829	*P* = 0.0009
	IL-2^{+}IL-17^{+}	-0.6333	*P* = 0.0002	IL-17^{+}	-0.5537	*P* = 0.0018
	TNF-α^{+}	-0.4473	*P* = 0.0150	IL-2^{+}IL-17^{+}	-0.4168	*P* = 0.0245
	TNF-α^{+}IL-2^{+}IL-17^{+}	-0.3242	*P* = 0.0862	IFN-γ^{+}IL-2^{+}	-0.3583	*P* = 0.0563
	TNF-α^{+}IL-2^{+}	-0.3242	*P* = 0.0862	IFN-γ^{+}TNF-α^{+}IL-17^{+}	-0.3497	*P* = 0.0629
	IFN-γ^{+}TNF-α^{+}IL-17^{+}	-0.2292	*P* = 0.2318	IFN-γ^{+}TNF-α^{+}	-0.3072	*P* = 0.1050
	IFN-γ^{+}IL-2^{+}	-0.1914	*P* = 0.7289	IFN-γ^{+}TNF-α^{+}IL-2^{+}IL-17^{+}	-0.1933	*P* = 0.3150
	TNF-α^{+}IL-17^{+}	-0.1906	*P* = 0.3220	TNF-α^{+}IL-2^{+}	-0.1476	*P* = 0.4450
	IFN-γ^{+}	-0.0676	*P* = 0.726	IL-2^{+}	-0.0976	*P* = 0.6146
	IL-2^{+}	-0.0399	*P* = 0.8372	TNF-α^{+}IL-17^{+}	-0.0266	*P* = 0.8910
	IFN-γ^{+}IL-2^{+}IL-17^{+}	-0.0309	*P* = 0.8735	TNF-α^{+}	0.1904	*P* = 0.3225
	IFN-γ^{+}TNF-α^{+}	0.0687	*P* = 0.7233	IFN-γ^{+}	0.6789	*P* < 0.0001

Supplementary Table 1. Correlations between protection level and vaccine-induced immune responses pre- and post-infection. Correlations between protection (CFU) and ESAT-6-specific T-cells are shown in the table. Spearman's r and *p* values of the correlations are indicated.
